# Plasma and urine neutrophil gelatinase-associated lipocalin as markers of acute kidney injury in critically ill adults

**DOI:** 10.1186/cc10958

**Published:** 2012-03-20

**Authors:** R Matsa, E Ashley, J Osypiw, V Sharma, A Walden, L Keating

**Affiliations:** 1 Oxford University Hospitals NHS Trust, Oxford, UK; 2 Royal Berkshire NHS Foundation Trust, Reading, UK

## Introduction

Acute kidney injury (AKI) has significant impact both on the morbidity and mortality in patients on the ICU. The current definition and classification of AKI [1] uses changes in both the serum creatinine and urine output. This occurs late in the evolution of AKI and so the diagnosis can be delayed. Early detection of AKI could allow earlier recognition and treatment of the condition. Neutrophil gelatinase-associated lipocalin (NGAL) is a 25-kDa protein produced in response to inflammation, infection and kidney injury [2] and is found in blood and urine samples obtained from patients soon after the onset of AKI [3]. Earlier studies have shown that NGAL can be detected as early as 2 hours following AKI [2]. The predictive value of NGAL in the ICU may help the earlier recognition of AKI. The aim of the study is to determine whether plasma and/or urine NGAL levels can predict the earlier incidence of AKI (as defined by RIFLE criteria) in critically ill patients.

## Methods

This single-centre prospective observational study is currently recruiting 200 consecutive adult patients with no AKI on presentation to the ICU. Serial samples of plasma and urine are collected on all patients included in the study at 0 hours and then every 24 hours in the ICU stay up to 72 hours and assayed for NGAL using a turbidimetric assay on the standardised automated analyser.

## Results

Results on the first 27 patients are currently available. The predictive performance of pNGAL at admission for AKI (24 hours prior to creatinine-based (RIFLE) diagnosis) was good (AUC-ROC 0.8 (95% CI 0.88 to 1.03)). The predictive performance of uNGAL at admission for the occurrence of AKI (24 hours prior to creatinine-based (RIFLE) diagnosis) (AUC-ROC 0.77 (95% CI 0.47 to 1.07)) was fair. See Table 1.

**Table 1:** Sensitivity and specificity of pNGAL (time '0) to diagnose AKI occurrence at 24 hours

Cut-off (ng/ml)	Sensitivity (95% CI)	Specificity (95% CI)
<270.0	82.6 (61.2 to 95)	100 (37.6 to 100)
<316.5	86.9 (66.4 to 97.2)	100 (37.6 to 100)
<381.0	91.3 (71.9 to 98.9)	100 (37.6 to 100)

## Conclusion

Early results on pNGAL suggest that it could be an independent predictor of AKI in an unselected population of critically ill adults. Further results are awaited.
